# Demographic census confirms a stable population of the critically-endangered caryophyllid coral *Polycyathus chaishanensis* (Scleractinia; Caryophyllidae) in the Datan Algal Reef, Taiwan

**DOI:** 10.1038/s41598-020-67653-8

**Published:** 2020-06-29

**Authors:** Chao-Yang Kuo, Shashank Keshavmurthy, Aichi Chung, Ya-Yi Huang, Sung-Yin Yang, Yei-Chia Chen, Chaolun Allen Chen

**Affiliations:** 1 Biodiversity Research Center, Academia Sinica, Nangang, Taipei, 115 Taiwan; 2 Shimoda Marine Research Center, University of Tsukuba, Shimoda, Shizuoka 415 Japan; 3 Institute of Oceanography, National Taiwan University, Daan, Taipei, 106 Taiwan; 4 Department of Life Science, Tung-Hai University, Xitun, Taichung, 404 Taiwan

**Keywords:** Ecology, Biodiversity, Marine biology, Environmental impact, Conservation biology

## Abstract

*Polycyathus chaishanensis* is a symbiotic caryophyllid coral described from a single population in a tidal pool off Chaishan, Kaohsiung, Taiwan. Due to its rarity, *P. chaishanensis* was declared a critically-endangered species under the Taiwan Wildlife Protection Act. In May 2017, a *P. chaishanensis* colony was discovered in the intertidal area of the Datan Algal Reef, Taoyuan, Taiwan. To determine whether this is a stable population in the algal reef, a demographic census—including data on occurrence, distribution, and colony size—was carried out in the algal reef in southern Taoyuan. Intertidal censuses and sediment collections were conducted at five different sections—Baiyu, Datan G1, Datan G2, Yongxing, and Yongan algal reefs—during the monthly spring low tide from July 2018 to January 2019. In total, 84 colonies—23 in Datan G1 and 61 in Datan G2—were recorded from a tidal range of − 160 to − 250 cm, according to the Taiwan Vertical Datum 2001 compiled by the Central Weather Bureau. No *P. chaishanensis* was found in Baiyu, Yongxing, or Yongan. The *P. chaishanensis* colony sizes ranged from 2.55 to 81.5 cm in diameter, with the larger *P. chaishanensis* present in the lower intertidal zone. Sediment was extremely high, with monthly site averages ranging from 3,818.26 to 29,166.88 mg cm^−2^ day^−1^, and there was a significant difference between sites and months, both of which affected the distribution of *P. chaishanensis* in the algal reef. Our study confirms the existence of a second population of *P. chaishanensis* in Taiwan, highlighting the importance of the Datan Algal Reef for the survival and protection of this critically-endangered caryophyllid coral and why it is so urgent that the reef should be conserved.

## Introduction

*Polycyathus chaishanensis* is a caryophylliid coral described from the coast of Chaishan, Kaohsiung, Taiwan^1^. The other known *Polycyathus* species are found in the Pacific and Atlantic Oceans from the sublittoral zone (> 12 m) to depths greater than 400 m^2–5^; *P. chaishanensis*, however, was discovered from a single population composed of fewer than 50 small encrusting colonies in a tidal pool (< 3 m) during high tide, which represents the shallowest *Polycyathus* recorded so far. This makes *Polycyathus,* including *P. chaishanensis,* a model genus for understanding the evolution of scleractinian corals, such as whether *Polycyathus* evolved from shallow to deep waters or vice versa. *P. chaishanensis* is the only one of the 22 known *Polycyathus* species^6^ associated with the Symbiodiniaceae algae *Cladocopium* C1^1^. *P. chaishanensis* was declared a critically-endangered coral species in May 2017, and is now protected by the Taiwanese Wildlife Act (TWA)^7^.

Apart from the taxonomic description, little is known about the baseline ecology or environment of *P. chaishanensis*. Reconnaissance surveys of the holotype locality in Chaishan showed that its environment is different from those of other tropical reef-building corals, which usually include clean water and relatively low sedimentation rates^8^. For example, the normal sedimentation rate of a healthy coral reef is around 10 mg cm^−2^ day^−1^ maximum^9^. Values above such a rate would cause some degree of reef degradation, and above 50 mg cm^−2^ day^−1^ would have catastrophic consequences^10^. The Chaishan coast is a sandy beach mixed with various sizes of carbonaceous rocks, which originated from nearby coastal hills made by an uplifted Pleistocene reef developed about 0.4–0.6 Mya^11^. The rock pool is highly turbid and the water column contains a high concentration of sand and particles formed by erosion, waves, and tide, which may have deterred most other scleractinians, even when a hard substratum is available^1^. *P. chaishanensis* was found on well-lifted reefs dominated by green and crustose coralline algae (CCA), suggesting that *P. chaishanensis* may prefer marginal habitats like this sandy rock pool.

**Figure 1:**
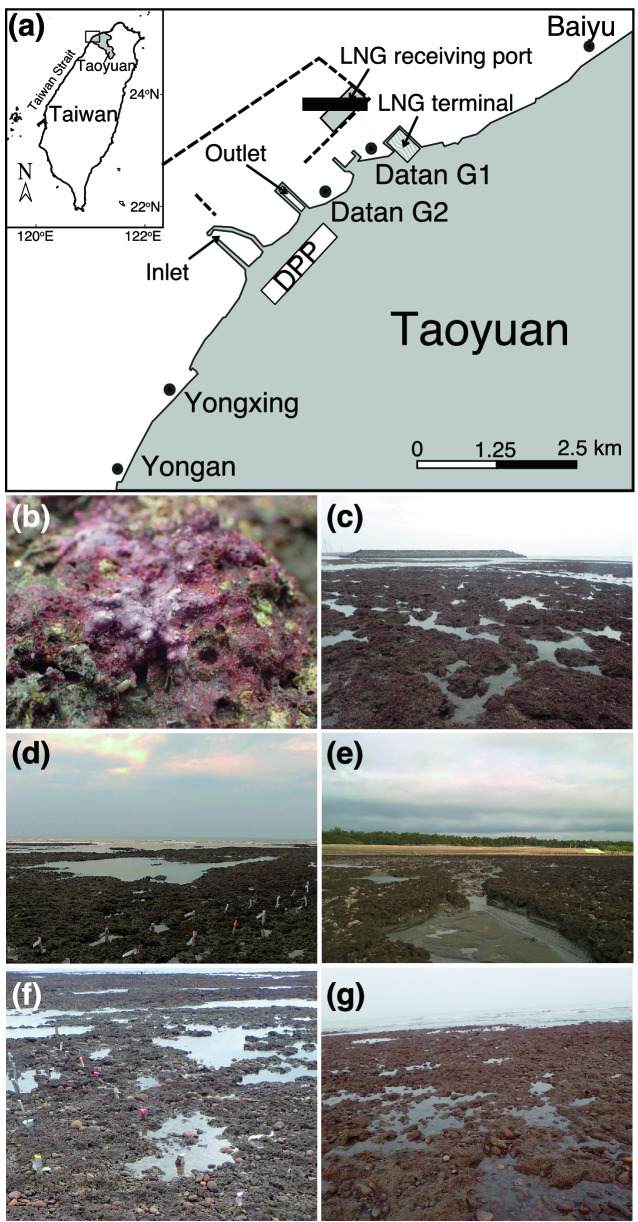
Algal reefs off the coast of southern Taoyuan, Taiwan. Map showing the study sites, including Baiyu, Datan G1, Datan G2, Yongxing, and Yongan (**a**). The Datan Power Plant (DPP) and planned construction range of the receiving port, embankment (dot lines), and liquefied natural gas (LNG) terminal are indicated. The algal reef is mainly composed of CCA (**b**), with porous algal reefs growing continuously (**c**,**d**), disrupted by sand flats (**e**), or mixed with cobbles (**f**,**g**). White line in (**b**) = 1 cm.

In May 2017, a single colony of *P. chaishanensis* was discovered in the intertidal area of the Datan Algal Reef, Taoyuan, Taiwan^12^ (Fig. 1a). This algal reef is unique to northwestern Taiwan, and is the only other habitat for *P. chaishanensis* in Taiwan. The algal reef is mainly made up of CCA (Fig. 1b), which occupies the intertidal flat towards the upper sublittoral zones along the 27-km coast of Taoyuan City; it represents the first monumental biotic reef in the western coast of Taiwan that also contains sandy and/or muddy habitats^13,14^. The porous algal reefs grow continuously (Fig. 1c,d), are disrupted by sand flats (Fig. 1e), or are mixed with cobbles (Fig. 1f,g), and host relatively high benthic diversity and biomass, such as crustaceans, polychaetes, and sipunculans^14^. The Taoyuan algal reef was thus proposed to be a “stepping stone” to connect reef-associated species between tropical coral reefs and non-reefal coral communities in the Taiwan Strait^15^.

To confirm whether there is a stable population of *P. chaishanensis* in the algal reef, we conducted a demographic census—surveying the occurrence, distribution, and colony size of *P. chaishanensis—*and measured the sedimentation rate on the algal reef in southern Taoyuan. Although the algal reef has historically extended 27 km along the coast off of Taoyuan City, 75% of the reef in northern Taoyuan has been degraded due to industrial pollution and habitat destruction^12^; the remaining 25% in the south includes the Baiyu, Datan, Yongxing, and Yongan reefs and are in better condition^16^. Nevertheless, development and construction of the industrial port and liquefied natural gas receiving and storage terminal continuously threaten the survival and integrity of the algal reef ecosystem^17^. Hence, action is urgently needed to conserve the algal reef in southern Taoyuan^12^. Confirming the population status of the critically-endangered species *P. chaishanensis* can help boost conservation efforts and prevent the local destruction of the algal reef in southern Taoyuan.

## Results

**Figure 2:**
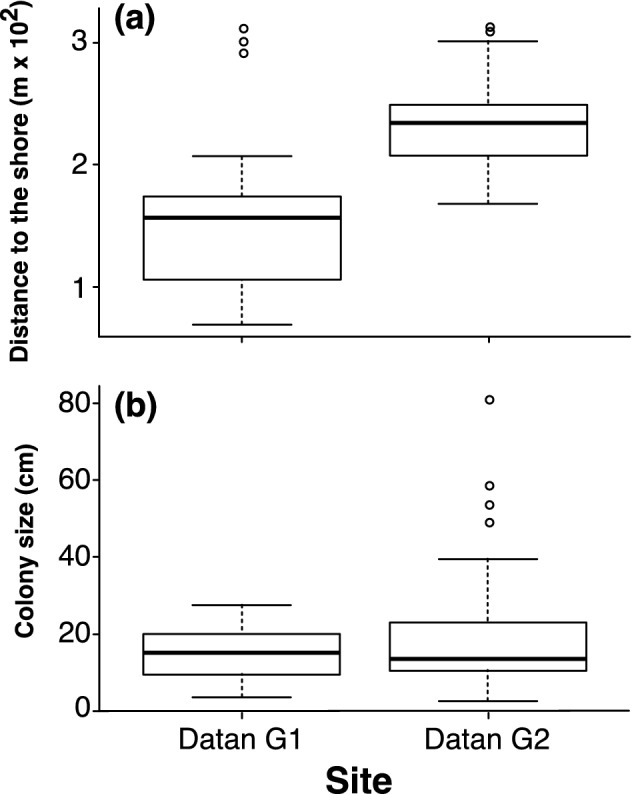
Box-plot of *Polycyathus chaishanensis* distance to the shore in the intertidal zone of Datan G1 and Datan G2 (**a**) and box-plot of *P. chaishanensis* colony sizes in Datan G1 and Datan G2 (**b**).

Eighty-four colonies of *P. chaishanensis* were recorded in this study. Among these, 21 were discovered 68.83 to 313.71 m from the shore in Datan G1, and 63 between 168.68 and 315.2 m in Datan G2. No *P. chaishanensis* colonies were recorded from the same range in the reefs in Baiyu, Yongxing, or Yongan after monthly surveys at low tide between July 2017 and February 2019. *P. chaishanensis* in Datan G1 was located significantly closer to the shore than those in Datan G2 (Mann–Whitney Test, *p* < 0.05, Fig. 2a).

**Figure 3:**
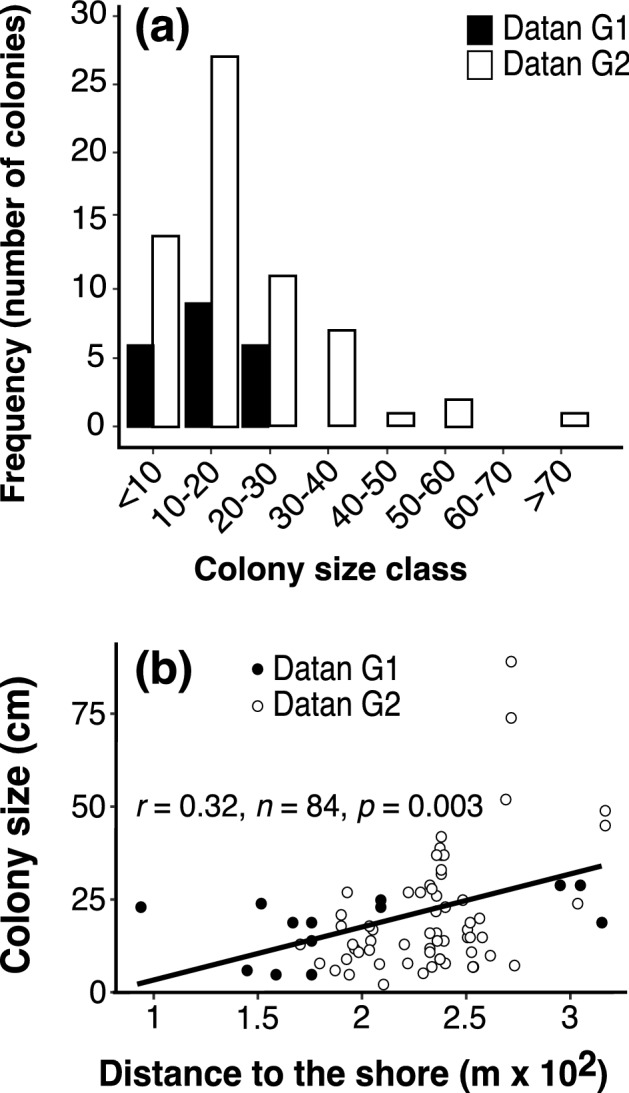
Colony size frequency of *Polycyathus chaishanensis* in Datan G1 and Datan G2 (**a**) and linear regression of the colony size (cm) with the distance to the shore. The open and closed dots indicate the colonies measured in Datan G1 and Datan G2, respectively (**b**).

*Polycyathus chaishanensis* colony sizes in the Datan Algal Reef ranged from 2.55 to 81.5 cm in diameter, with a mean of 17.97 cm. The mean colony size of *P. chaishanensis* in Datan G1 (14.786 ± 1.450 cm, n = 21) (mean ± SE) was not significantly different from that of Datan G2 (19.035 ± 1.818, n = 63) (Student *t*-test = − 1.098, *p* = 0.279, Fig. 2b). Although there were several large colonies (> 50 cm) recorded in Datan G2, the size frequency was not significantly different between these two sites (*X*^2^-test = 4.894, *p* = 0.558, Fig. 3a). However, linear regression showed a significant correlation between colony size and distance to the shore, suggesting that the larger *P. chaishanensis* tended to occur at lower intertidal areas (F_(1,82)_ = 9.684, *p* < 0.05, R^*2*^ = 0.106, Fig. 3b).

**Figure 4:**
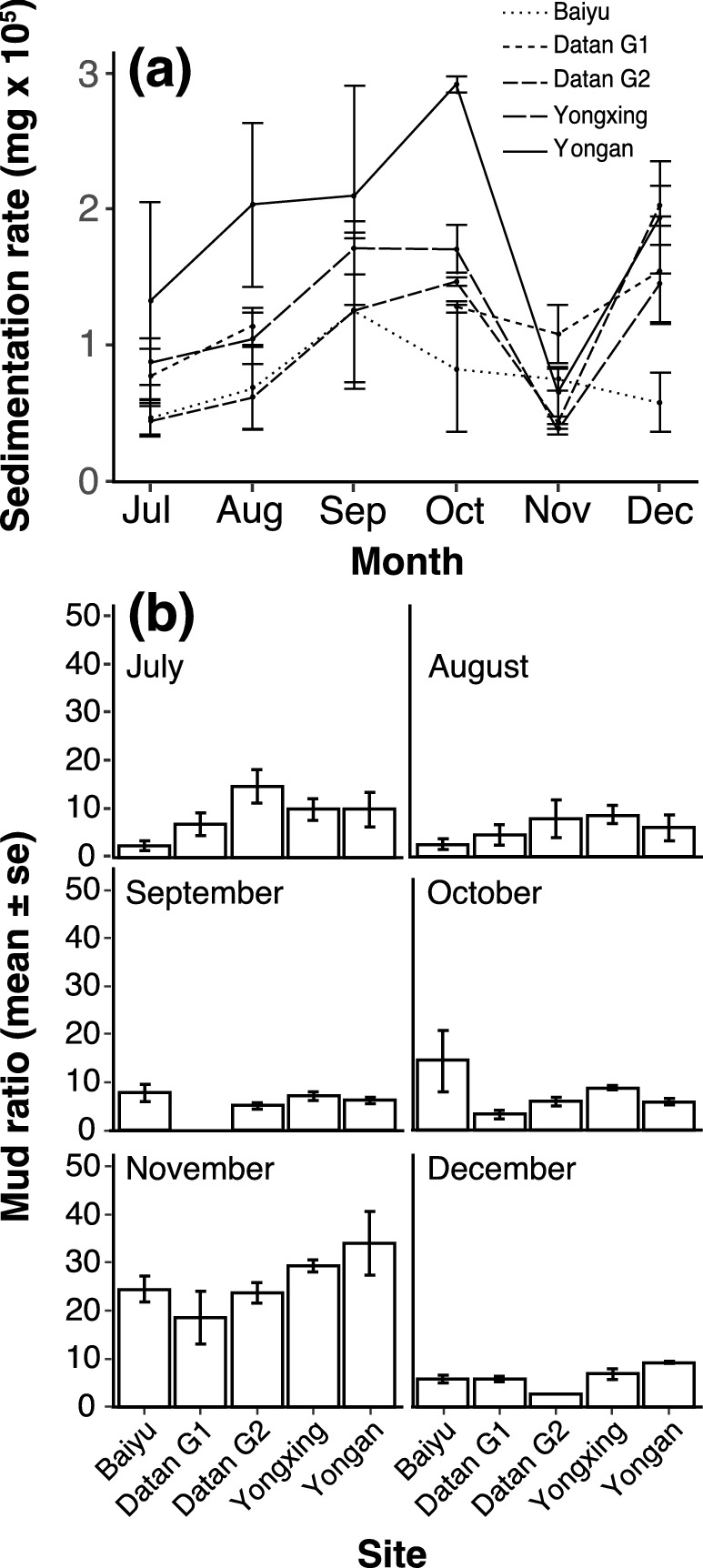
Daily average dry weight (mg, mean ± SE) of total sediment per cm^2^ (**a**) and monthly mud ratio (mean ± SE) (**b**) at five studied sites from July to December, 2018. All three sediment traps placed in Datan G1 were lost in September due to strong waves.

**Figure 5:**
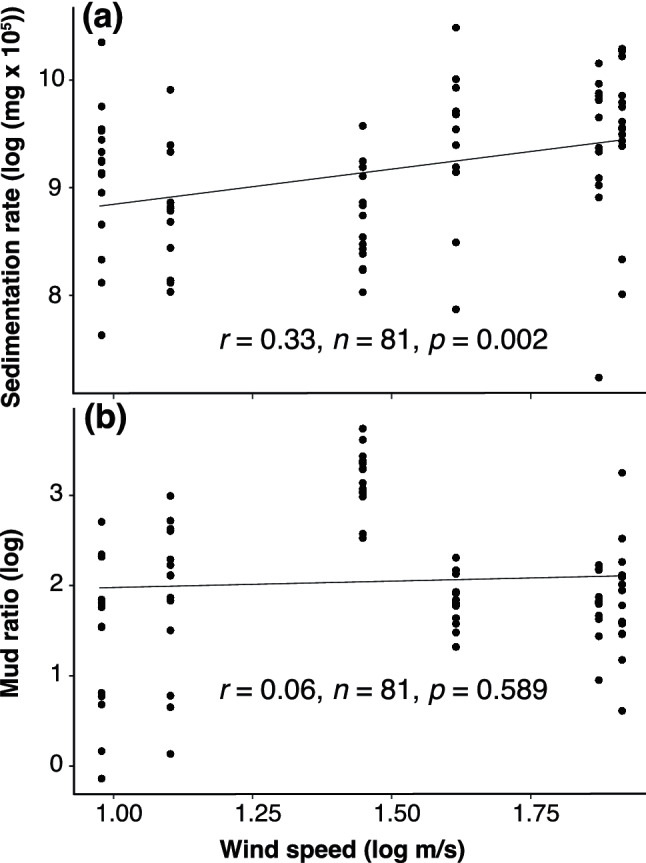
Linear regression of the sedimentation rate (**a**) and mud ratio (**b**) with wind speed. Each dot represents a sediment trap.

Throughout the study period, the sedimentation rate (mud and sand) in individual traps ranged from 1,416.56 to 36,592.10 mg cm^−2^ day^−1^ (see Supplementary Table S1 online), while monthly site averages ranged from 3,818.26 to 29,166.88 mg cm^−2^ day^−1^ (Fig. 4a). The best-fitting model (GLMM) to account for the variation in the mud ratio was the full model, which included site and month (AICc = 156.27, *df* = 31) as opposed to month (AICc = 171.74, *df* = 8) or site (AICc = 172.12, *df* = 7) alone. The sedimentation rate was significantly different across sites (F = 7.873, *p* < 0.001) and months (F = 7.321, *p* < 0.001, Table 1), and there was no significant interaction between site and month (F = 1.418, *p* = 0.159). Sedimentation rate was significantly correlated with wind speed (Fig. 5a).

**Table 1:** The separated ANOVA results for sedimentation rate and mud ratio based on the best fitting generalized linear mixed model (GLMM): mud ratio ~ site × month.

	df	Sum of squares	Mean square	F	*p*
**Sedimentation rate**					
Site	4	8.604	2.151	7.873	** < 0.0001**
Month	5	10.000	2.000	7.321	** < 0.0001**
Site × month	19	7.360	0.387	1.418	0.159
**Mud ratio**					
Site	4	2.386	0.597	3.262	0.018
Month	5	21.769	4.354	23.818	** < 0.0001**
Site × month	19	6.975	0.367	2.008	0.024

Significant effects (*p* < 0.01) shown in bold.

The mud ratio was applied to reveal the spatial and temporal contributions of mud and sand to the total sediment, calculated as the weight of mud vs weight of total sediment. The lowest and highest mean mud ratios were found in Baiyu in August (2.57%) and Yongan in November (34.09%), respectively (Fig. 4b). The best-fitting model to account for the variation in mud ratios was the full model (AICc = 118.32, *df* = 31) as opposed to month (AICc = 128.65, *df* = 8) or site (AICc = 183.4, *df* = 7) alone. The mud ratios were highly significantly different across the months (F = 22.818, *p* < 0.001), but not among sites (F = 3.262, *p* = 0.018) or the interaction between site and month (F = 2.008, *p* = 0.024) (Table 1). The mud ratio at each site in November was 2.92–4.43-fold higher than the average mud ratio for the other months (Fig. 4b). The mud ratio was not affected by wind speed during the study period (Fig. 5b).

## Discussion

Baseline ecology and environmental data are urgently needed to accurately illustrate the conservation status of *P. chaishanensis* and predict how further development of a liquefied natural gas receiving terminal will affect this status^17^. Our demographic census confirmed the occurrence of *P. chaishanensis* in the Datan Algal Reef, southern Taoyuan, Taiwan. Both the number of coral colonies (84 in total) and colony size indicate that *P. chaishanensis* forms a stable population in the Datan Algal Reef, reiterating the necessity for conservation action.

Our study confirmed the previous finding that *P. chaishanensis* mostly inhabits shallow waters^1^. All *Polycyathus* species, except *P. chaishanensis* and *P. fulvus*^18^, are azooxanthellate, and *P. fulvus* (30–50 m), as well as all *Polycyathus* species except *P. chaishanensis*, are found in deep waters; e.g., *P. mullerae* contributes to the formation of the mesophotic reef at 55 m deep in the Mediterranean^19^. *P. mayae* and *P. senegalensis* are found at 127–309 and 12–143 m deep, respectively, in the Gulf of Mexico^5^. In Colombia, both species are recorded from 75–217 and 73–152 m deep, respectively^4^. At the holotype locality in Chaishan, Kaohsiung, *P. chaishanensis* is distributed in an intertidal pool estimated to be less than 300 cm deep during high tide and − 50 cm during low tide, according to the Taiwan Vertical Datum 2001 (TWVD 2001). *P. chaishanensis* was found at a similar tidal level in the Datan Algal Reef (below − 160 cm), confirming that *P. chaishanensis* can adapt to a shallow-water environment with high turbidity and a water column with a high concentration of sand and particles due to erosion, waves, and tide^1^.

We hypothesized that, unlike other *Polycyathus* species*, P. chaishanensis* has adapted to shallow tidal pools or the intertidal area and is symbiotic with the Symbiodiniaceae *Cladocopium* (C1). Corals in disturbed environments tend to associate with stress-tolerant symbionts in the genus *Durusdinium* and some *Cladocopium* species (C3 and C15). In contrast, corals from highly sedimented environments in Hong Kong and Singapore were also found to associate with *Cladocopium* C1^20,21^. The reason it associates with *Cladocopium* C1—even in environments with high sediment—is that, although stress tolerant, *Durusdinium* sp. is considered a selfish genus that does not provide efficient nutrient translocation to its coral host. However, Baker et al.^22^ showed that *Cladocopium* sp. might be involved in efficient nitrogen assimilation and carbon translocation to the hosts. On the other hand, high turbidity might provide protection from light stress (elevated levels of irradiance), thereby reducing the impact of thermal bleaching events^20,23^. Highly turbid environments might also help corals obtain nutrients in the form of suspended particulate matter, thereby making the coral less dependent on the symbiont (irrespective of the type of symbiont) for its energy requirements and allowing it to exist in shallow and turbid environments.

*Polycyathus chaishanensis* in the Datan Algal Reef was distributed in the lower intertidal zone, and larger colonies were found towards the sublittoral zone. It was suggested that two abiotic gradients—disturbances from wave damage and light availability—determine the distribution and growth of reef-building corals in tropical waters^24,25^ and species diversity in the rocky intertidal zone^26,27^. Similar abiotic factors plus substratum availability can explain the distribution pattern of *P. chaishanensis* in the Datan Algal Reef. The algal reef stretches along the intertidal sand flat towards the sublittoral zone along the Taoyuan coast, and the Datan Algal Reef its best-developed section^14^ (Fig. 1). High sedimentation rate caused by strong winds and waves could easily bury the reefs in the upper intertidal zone. Thus, even if new *P. chaishanensis* juveniles could settle, they would also be buried. By contrast, in the lower intertidal zone, particularly in Datan G1 and Datan G2, the algal reef structure is more continuous and not interrupted by sand or cobbles (Fig. 1c,d), and small grooves or caves on the porous algal reef provide shelter for *P. chaishanensis* to be submerged underwater during low tide. In addition, the monthly spring low tide occurs at midnight, early morning, late afternoon, or after sunset along the Taoyuan coast, suggesting that *P. chaishanensis* prefers tidal zones less affected by desiccation from sun exposure (see Supplementary Fig. S1 online). These scenarios were also found to support several stress-tolerant scleractinian coral species at a similar tidal range—*Porites* sp., *Goniopora* sp., *Pseudosiderastrea tayami*, and *Oulastrea crispata*^14^.

The sedimentation rate was significantly correlated with wind speed on the intertidal algal reef in Taoyuan. Coral communities in the fringing reefs of Magnetic Island, northeastern Australia are also influenced by wind regimes, and the height of locally produced, short-period wind waves control the magnitude of near-bed suspended sediment concentrations^28^. The sedimentation rate^9^ in a healthy coral reef is around 10 mg cm^−2^ day^−1^, and the rate in the worst case scenario of coral degradation^10^ is estimated to be above 50 mg cm^−2^ day^−1^. Although sedimentation rates vary depending on the method and the sediment collected by sediment traps seems to be around 1/3 of in nature habitats^29^, the sedimentation rate in the algal reef ranges from 3,818.26 to 29,166.88 mg cm^−2^ day^−1^ and is over 300-fold the minimum in a coral reef. Hence, it is clear that, except for *P. chaishanensis* and a handful of stress-tolerant species, no corals can survive in an environment with so much sediment or build a coral-dominant reef structure. In contrast, crustose coralline algae (CCA) might be more tolerant to sand scouring or burial in a high sediment environment^30,31^. A study using the CCA collected at rocky sites along the northwestern coast of the USA showed that CCA can survive lengthy anoxic burial for three months with only slightly discoloration^32^. A similar experimental approach can be conducted to examine how long CCA can survive in the Datan Algal Reef under sand scouring or burial conditions. Furthermore, it is vital to understand how burial conditions affect the growth rate of CCA, the fundamental reef builder in the algal reef.

In conclusion, our study confirms the existence of a second population of *P. chaishanensis* in Taiwan in the Datan Algal Reef, which has proven to be an unprecedented ecosystem not only for Taiwan, but around the world^12,14,33^. The *P. chaishanensis* population in Datan is the only existing healthy and stable population in Taiwan, since the holotype locality of *P. chaishanensis*, Chaishan, was destroyed by coastal development in Kaohsiung^34^. In addition, the recent discovery of an unexpected large colony of *P. chaishanensis* (110 cm long and 80 cm wide) at the − 250 cm tidal level in Datan G2 confirms the urgent need for conservation action^35^, including measures that allow the public to participate in decisions involving energy-related construction issues and better advising around the government’s policy decisions^36^. Unfortunately, the Datan Algal Reef is currently facing destruction from the development and construction of liquefied natural gas (LNG) receiving and storage terminals and ports^17^, which also severely threaten the survival of the merely extant local population of *P. chaishanensis*. For example, there are at least seven colonies of *P. chaishanensis* located within 200 m of the scheduled trestle bridge in the LNG terminal in Datan G1, with the closest one being only 52 m away (see Supplementary Table S2, Fig. S2 online). The Datan Algal Reef has been designated a Hope Spot by the marine conservation NGO Mission Blue^33^, and the overall strength, willingness to pay (WTP), for protecting the algal reef is higher than for the gas receiving station in Taiwan^36^; nonetheless, both the Datan Algal Reef and *P. chaishanensis* face regional extinction if serious conservation action is not taken against coastal development.

## Methods

Field surveys were conducted monthly during the spring low tide from June 2017 to February 2019 in the southern sections of the Taoyuan algal reef, including Baiyu, Datan, Yongxing, and Yongan (Fig. 1a). These sections were chosen based on the townships nearby. The Datan Algal Reef was divided into two subsections, Datan G1 and Datan G2, based on an environmental impact assessment report for the construction of a liquefied natural gas terminal/storage project by the Taiwan Chinese Petrol Corporation (CPC), which provides fuel to the nearby Datan liquefied natural gas (LNG) power plant (Fig. 1a). The algal reef, predominantly built by stacks of crustose coralline algae, occupies the intertidal sand flats during low tide and extends towards the sublittoral zone at five meters deep^12,14^. When exposed to the air during the low tide, the reef can be classified as a continuous and porous structure (Fig. 1b,c), disrupted by sand flats (Fig. 1d) or mixed with cobbles (Fig. 1e,f).

Due to turbid waters, it was not possible to use SCUBA or snorkeling to survey *P. chaishanensis* occurrence or measure its colony sizes. Thus, we conducted intertidal reef walking on each algal reef section and subsection parallel to the coastline during monthly spring low tides. Preliminary surveys indicated that *P. chaishanensis* did not occur at tidal heights greater than − 160 cm, according to the Taiwan Vertical Datum 2001 (TWVD) compiled by the Central Weather Bureau. This − 160 cm tidal level is also the most common monthly low tide level, except during the extreme spring tides at the end of the year. We therefore conducted the surveys at the − 160 to − 250 cm tidal range. *P. chaishanensis* was identified in situ, and the colony locality was recorded by a GPSMAP 64st and mapped onto Google Earth to measure the horizontal distance to the scheduled trestle bridge of the LNG terminal (see Supplementary Fig. S2, Table S2 online). Colony size was determined by measuring the maximum length (L) and maximum width (W), which is perpendicular to the length, with the formula (L + W)/2.

Two indices—the distribution pattern, in terms of the distance of each colony to the shore, and the colony size frequency distribution—were utilized to compare the characteristics of *P. chaishanensis* found in different algal reef sections*.* To examine the impact of sedimentation on the occurrence and distribution of *P. chaishanensis* in different algal reef sections, sediment at each site was collected using PVC cylindrical sediment traps (5 cm in diameter and 16 cm in depth, with metal mesh in the opening mouth) modified from the design described in English (1997)^37^. Each sediment trap was attached with a star picket for deploying the trap and maintaining it around 30–40 cm above the bottom. Three sediment trap replicates were deployed at the − 160 cm tide level at low tide in each reef section for 48 h to cover two full tidal cycles during the monthly spring tide period from July to December, 2018. Sediment from each trap was rinsed with distilled water to remove salts, filtered through a 100 μm filter, and air dried in an oven at 60 °C for 48 h before weight measurement. Sediment larger than 100 μm in diameter was defined as “sand,” while the rest (smaller than 100 µm) was defined as “mud.” The mud ratio—weight of mud divided by weight of total sediment (mud plus sand)—was calculated to examine the contribution of sand and mud to water quality across the five algal reef sections.

The distribution pattern, in terms of the distance of each colony to the shore, between Datan G1 and Datan G2 populations was compared using the Mann–Whitney test. To test for significant difference in *P. chaishanensis* colony sizes between the two populations, colony size was first converted into size class with a 10-cm interval, then Pearson’s chi-square test was applied. Yate’s continuity correlation^38^ was also applied due to the presence of size classes with fewer than five individuals. A Student’s t-test was used to test for significant difference in mean colony size between the two populations. Normality and homogeneity of variance were tested using the Shapiro–Wilk test^39^ and Levene’s test^40^, and log (x + 1) data transformation was applied when necessary.

A generalized linear mixed model (GLMM) was used to explore the spatial and temporal variation in the dry weight of sediment and mud ratio in sediment traps, separately. The full model included fixed effects of the site (five levels: Baiyu, Datan G1, Datan G2, Yongxing, and Yongan) and month (six levels: from June to December). We then compared three alternative models—(i) mud ratio ~ Site, (ii) mud ratio ~ Month, (iii) mud ratio ~ Site × Month—using Akaike information criterion (AIC). In all cases, we included sediment traps as a random effect (~ 1|Trap) to account for intra-site differences of mud ration in the sediment trap level. Kruskal–Wallis test was used to determine the spatial variation of the mud ratio for each month and temporal variation within each site. The *lme4* (v. 1.1.19) and *lmerTest* (v. 3.1.0) packages in R-3.4.4 were applied^41^.

## Supplementary information

Supplementary file1 (DOCX 345 kb)

## Data Availability

All additional data from the research are provided in “Supplementary Information”.
