# Supplementary material for: Demographic census confirms a stable population of the critically-endangered caryophyllid coral Polycyathus chaishanensis (Scleractinia; Caryophyllidae) in the Datan Algal Reef, Taiwan
Source: Sci Rep. 2020 Jun 29;10:10585. doi: 10.1038/s41598-020-67653-8 (PMC7324372; doi:10.1038/s41598-020-67653-8)
Supplement: Supplementary file 1 — Supplementary file1 (DOCX 345 kb) [file 41598_2020_67653_MOESM1_ESM.docx]

**Demographic census confirms a stable population of the critically-endangered caryophyllid coral *Polycyathus chaishanensis* (Scleractinia; Caryophyllidae) in the Datan algal reef, Taiwan**

Chao-Yang Kuo^1^, Shashank Keshavmurthy^1^, Aichi Chung^1^, Ya-Yi Huang^1^, Sung-Yin Yang^1,2^, Yei-Chia Chen^1^, Chaolun Allen Chen^1,3,4*^

^1^Biodiversity Research Center, Academia Sinica, Nangang, Taipei 115, Taiwan

^2^Shimoda Marine Research Center, University of Tsukuba, Shimoda, Shizuoka 415, Japan

^3^Institute of Oceanography, National Taiwan University, Daan, Taipei 106, Taiwan

^4^Department of Life Science, Tung-Hai University, Xitun, Taichung 404, Taiwan

*: Corresponding author, e-mail: cac@gate.sinica.edu.tw

Table S1. The dry weight (mg cm^-2^ d^-1^) of mud and sand collected from each sedimentation trap in this study. “-” indicates lost sample.

|  |  | July | | August | | September | | October | | November | | December | |
| --- | --- | --- | --- | --- | --- | --- | --- | --- | --- | --- | --- | --- | --- |
| Site | Replicate | Mud | Sand | Mud | Sand | Mud | Sand | Mud | Sand | Mud | Sand | Mud | Sand |
| Baiyu |  |  |  |  |  |  |  |  |  |  |  |  |  |
|  | 1 | 141.95 | 7,074.65 | 37.80 | 4,199.75 | 1,951.59 | 20,707.26 | 136.56 | 2,935.03 | 1,974.01 | 7,222.42 | 474.90 | 7,083.82 |
|  | 2 | 40.08 | 3,379.36 | 164.44 | 3,257.07 | 120.25 | 2,550.83 | 1,122.04 | 3,127.64 | 1,920.00 | 4,467.77 | 564.59 | 7,892.99 |
|  | 3 | 160.61 | 3,326.37 | 261.22 | 12,639.92 | 1,265.73 | 11,015.54 | 2,229.81 | 15,267.26 | 1,556.18 | 5,467.52 | 61.15 | 1,355.41 |
| Datan G1 | |  |  |  |  |  |  |  |  |  |  |  |  |
|  | 1 | 399.18 | 4329.17 | - | 10,640.39 | - | - | 404.59 | 11,774.78 | 3,120.51 | 7,454.78 | 603.31 | 10,950.32 |
|  | 2 | 257.61 | 11,277.45 | 218.72 | 9,265.61 | - | - | 256.82 | 13,341.15 | 923.31 | 6,283.82 | - | - |
|  | 3 | 699.68 | 6219.62 | 909.71 | 13,117.76 | - | - | 635.92 | 12,123.31 | 1,974.01 | 12,716.43 | 1,236.18 | 18,187.01 |
| Datan G2 | |  |  |  |  |  |  |  |  |  |  |  |  |
|  | 1 | 566.59 | 6,102.42 | 1,611.41 | 8,894.68 | 262.93 | 4,713.38 | 878.47 | 13,574.52 | 1,065.99 | 2,781.15 | - | 18,828.03 |
|  | 2 | 488.48 | 2,653.76 | 46.56 | 2,054.52 | 1,415.54 | 21,252.48 | 674.65 | 14,617.07 | 918.22 | 2,973.76 | 576.82 | 21,149.55 |
|  | 3 | 714.01 | 2,780.13 | 349.48 | 5,522.05 | 384.20 | 9,631.59 | 1,096.56 | 13,203.57 | 1,059.87 | 4,167.13 | - | - |
| Yongxing | |  |  |  |  |  |  |  |  |  |  |  |  |
|  | 1 | 968.41 | 5,861.91 | 843.78 | 7,052.76 | 1,350.32 | 19,575.03 | 1,616.31 | 1,7839.49 | 992.61 | 2,138.09 | 1,151.59 | 17,516.43 |
|  | 2 | 684.55 | 6,523.31 | 1,484.42 | 12,765.94 | 859.11 | 13,361.53 | 1,334.01 | 12,225.22 | 1,126.11 | 2,723.06 | 1,428.79 | 14,457.07 |
|  | 3 | 786.97 | 11,506.75 | 445.97 | 8,905.09 | 1,467.52 | 14,872.87 | 1,551.08 | 16,701.15 | 1,228.03 | 3,246.88 | 491.21 | 8,555.41 |
| Yongan | |  |  |  |  |  |  |  |  |  |  |  |  |
|  | 1 | 1,359.58 | 19,177.58 | 1,994.08 | 29,930.61 | 1,814.01 | 34,778.09 | 2,110.57 | 27,311.08 | 2,112.61 | 7,897.07 | - | 19,901.15 |
|  | 2 | - | - | 139.46 | 11,394.00 | 1,187.26 | 15,637.20 | 1,421.66 | 26,579.36 | 1,782.42 | 2,900.38 | 2,383.69 | 23,868.54 |
|  | 3 | 834.13 | 5,187.26 | 1,844.07 | 15,737.48 | 663.44 | 8,884.59 | 1,492.99 | 28,584.97 | 2,111.59 | 2,788.28 | 1,138.34 | 10,848.41 |

Table S2. GPS coordinates (WGS 84) and colony maximum length (L) and width (W) for *Polycyathus chaishanensis* colonies found in this study.

| Colony ID | Site | Latitude | Longitude | L (cm) | W (cm) |
| --- | --- | --- | --- | --- | --- |
| G144** | Datan G1 | 25.045460 | 121.057563 | 20 | 8 |
| G145 | Datan G1 | 25.045223 | 121.056456 | 20 | 18 |
| G146 | Datan G1 | 25.045142 | 121.056364 | 6 | 5 |
| G147 | Datan G1 | 25.045093 | 121.056369 | 25 | 15 |
| G148 | Datan G1 | 25.045087 | 121.056366 | 14 | 13 |
| G1139 | Datan G1 | 25.044957 | 121.055979 | 7 | 6 |
| G103 | Datan G1 | 25.044349 | 121.054865 | 24 | 18 |
| 80 | Datan G1 | 25.044310 | 121.054810 | 20 | 13 |
| 81 | Datan G1 | 25.044160 | 121.054930 | 17 | 16 |
| 82 | Datan G1 | 25.044510 | 121.055080 | 11 | 8 |
| 83 | Datan G1 | 25.044500 | 121.055130 | 12 | 7 |
| 90 | Datan G1 | 25.044520 | 121.055120 | 4 | 3 |
| 91 | Datan G1 | 25.044870 | 121.055850 | 14 | 14 |
| 92 | Datan G1 | 25.045300 | 121.056470 | 15 | 15 |
| 143*** | Datan G1 | 25.045766 | 121.057572 | 24 | 17 |
| 144*** | Datan G1 | 25.045766 | 121.057566 | 26 | 15 |
| 152* | Datan G1 | 25.046709 | 121.058758 | 30 | 20 |
| 153* | Datan G1 | 25.046901 | 121.059818 | 30 | 25 |
| 157* | Datan G1 | 25.046974 | 121.059489 | 20 | 15 |
| 158 | Datan G1 | 25.045402 | 121.057140 | 15 | 6 |
| 159* | Datan G1 | 25.045396 | 121.057143 | 6 | 4 |
| 74 | Datan G2 | 25.038436 | 121.048597 | 14 | 7 |
| 75 | Datan G2 | 25.038574 | 121.048642 | 9 | 4 |
| 76 | Datan G2 | 25.038662 | 121.048549 | 28 | 16 |
| 77 | Datan G2 | 25.038664 | 121.048585 | 10 | 10 |
| 78 | Datan G2 | 25.038771 | 121.048523 | 18 | 8 |
| 79 | Datan G2 | 25.038899 | 121.048774 | 15 | 11.5 |
| 80 | Datan G2 | 25.038769 | 121.048684 | 13 | 10 |
| 81 | Datan G2 | 25.038801 | 121.048709 | 12 | 11.5 |
| 82 | Datan G2 | 25.038798 | 121.048756 | 14 | 11 |
| 83 | Datan G2 | 25.038183 | 121.047660 | 7 | 4 |
| 95 | Datan G2 | 25.038686 | 121.048728 | 12 | 7 |
| 34 | Datan G2 | 25.039622 | 121.049063 | 11 | 7 |
| 96 | Datan G2 | 25.038883 | 121.048658 | 8.8 | 4 |
| 97 | Datan G2 | 25.038856 | 121.048592 | 3.3 | 1.8 |
| 98 | Datan G2 | 25.039246 | 121.048062 | 8.4 | 5 |
| 99 | Datan G2 | 25.039680 | 121.049028 | 11 | 10 |
| 100 | Datan G2 | 25.039708 | 121.049498 | 24 | 10 |
| 101 | Datan G2 | 25.039717 | 121.049475 | 9 | 5.6 |
| 102 | Datan G2 | 25.039281 | 121.048984 | 6.3 | 5.7 |
| 103 | Datan G2 | 25.037816 | 121.046806 | 12.5 | 10 |
| 104 | Datan G2 | 25.037659 | 121.046743 | 19 | 13 |
| 105 | Datan G2 | 25.037649 | 121.046754 | 22 | 5 |
| 106 | Datan G2 | 25.038349 | 121.046916 | 26 | 14 |
| 108 | Datan G2 | 25.038367 | 121.046893 | 16 | 16 |
| 109 | Datan G2 | 25.038363 | 121.046894 | 18 | 13 |
| 110 | Datan G2 | 25.038396 | 121.046904 | 20 | 7 |
| 111 | Datan G2 | 25.038385 | 121.046905 | 16 | 9 |
| 112 | Datan G2 | 25.038399 | 121.046923 | 15 | 8 |
| 113 | Datan G2 | 25.038405 | 121.046916 | 8 | 7 |
| 114 | Datan G2 | 25.038410 | 121.046935 | 14 | 8 |
| 115 | Datan G2 | 25.038411 | 121.046874 | 24 | 21 |
| 116 | Datan G2 | 25.038431 | 121.046881 | 16 | 14 |
| 117 | Datan G2 | 25.038291 | 121.046976 | 33 | 28 |
| 118 | Datan G2 | 25.038278 | 121.046980 | 34 | 24 |
| 119 | Datan G2 | 25.038274 | 121.046982 | 38 | 32 |
| 120 | Datan G2 | 25.038281 | 121.046982 | 43 | 25 |
| 121 | Datan G2 | 25.038289 | 121.047010 | 40 | 34 |
| 122 | Datan G2 | 25.038307 | 121.047010 | 38 | 30 |
| 123 | Datan G2 | 25.038292 | 121.047009 | 11 | 10 |
| 124 | Datan G2 | 25.038300 | 121.047017 | 15 | 13 |
| 125 | Datan G2 | 25.038290 | 121.047016 | 10 | 8 |
| 126 | Datan G2 | 25.038287 | 121.047023 | 38 | 31 |
| 127 | Datan G2 | 25.038281 | 121.047031 | 27 | 17 |
| 128 | Datan G2 | 25.038272 | 121.047010 | 17 | 13 |
| 129 | Datan G2 | 25.038264 | 121.046997 | 15 | 12 |
| 130 | Datan G2 | 25.038274 | 121.047020 | 24 | 23 |
| 131 | Datan G2 | 25.038260 | 121.047031 | 8 | 5 |
| 132 | Datan G2 | 25.038258 | 121.047026 | 30 | 15 |
| 133 | Datan G2 | 25.038247 | 121.047031 | 17 | 15 |
| 134 | Datan G2 | 25.038256 | 121.047026 | 13 | 9 |
| 135 | Datan G2 | 25.038262 | 121.047040 | 29 | 22 |
| 136 | Datan G2 | 25.038246 | 121.047033 | 12 | 10 |
| 137 | Datan G2 | 25.038402 | 121.047533 | 28 | 19 |
| 138 | Datan G2 | 25.038422 | 121.047540 | 9 | 7 |
| 139 | Datan G2 | 25.038992 | 121.047676 | 75 | 43 |
| 140 | Datan G2 | 25.039013 | 121.047680 | 90 | 73 |
| 141 | Datan G2 | 25.039092 | 121.047884 | 55 | 53 |
| 93 | Datan G2 | 25.039255 | 121.047570 | 25 | 20 |
| 94 | Datan G2 | 25.038248 | 121.047274 | 14 | 13 |
| 2019_96 | Datan G2 | 25.038015 | 121.046686 | 28 | 26 |
| 163 | Datan G2 | 25.038010 | 121.047050 | 19 | 12 |
| 2019_97 | Datan G2 | 25.039212 | 121.047220 | 46 | 33 |
| 2019_98 | Datan G2 | 25.039192 | 121.047202 | 50 | 49 |

*: colony is within 200 m of the LNG trestle bridge (see Figure 2S)

**: colony is within 100 m of the LNG trestle bridge (see Figure 2S)

***: colony is within 60 m of the LNG trestle bridge (see Figure 2S)


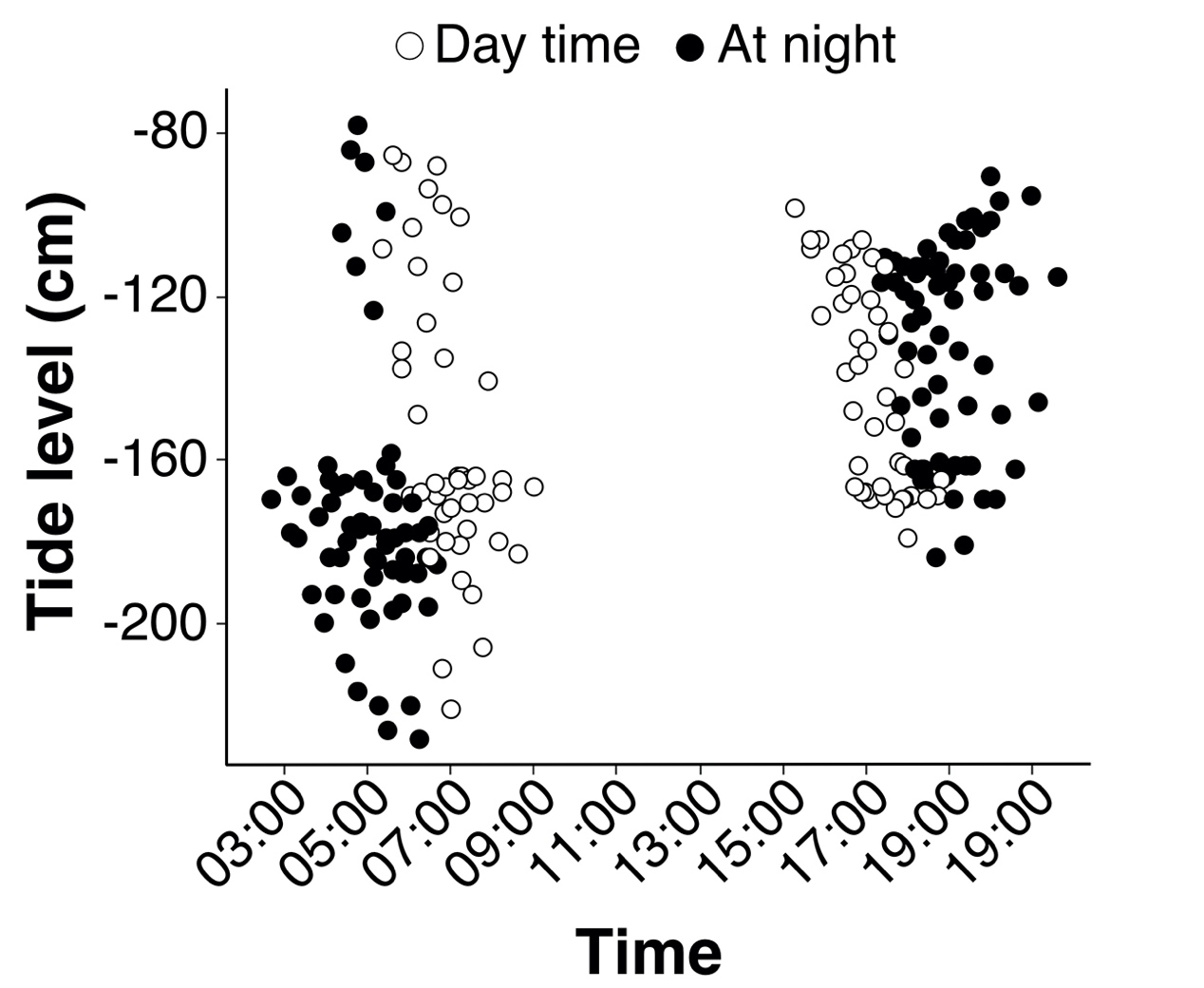


Figure S1. The tide level at low tide and the time of the low tide during the day of spring tides from March 1, 2018 to March 31, 2019. These data include at least one low tide lower than “-160 cm” during the day. White dots indicate the low tide after sunrise and before sunset. Black dots indicate the low tide during the rest of the day. Data were collected from the annual tidal forecast published by the Central Weather Bureau, Taiwan.


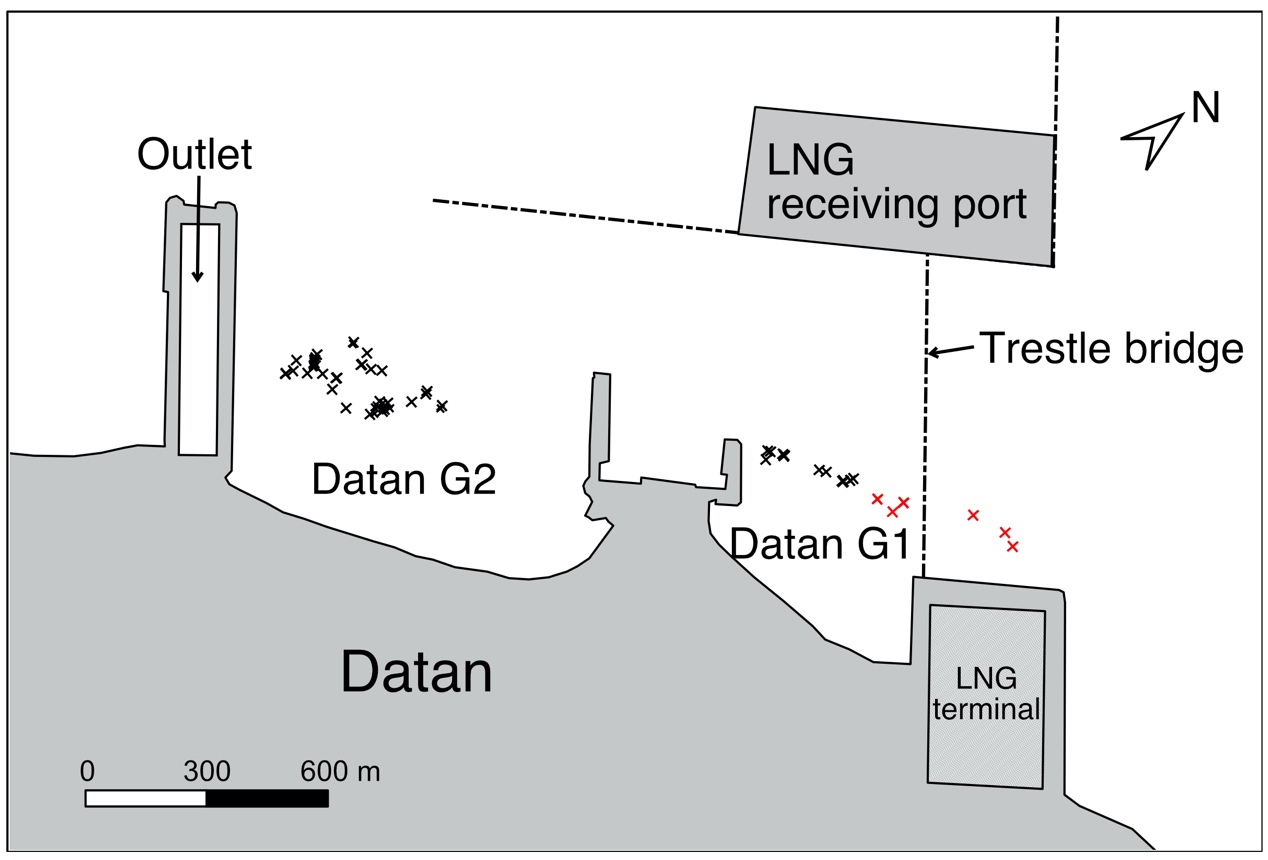


Figure S2. GPS locations of every *Polycyathus chaishanensis* colony in the Datan G1 and G2 algal reefs. “x” represents the location of each colony recorded. The red indicates the seven colonies located within 200 m of the trestle bridge.
